# HED LANG – A Hierarchical Event Descriptors library extension for annotation of language cognition experiments

**DOI:** 10.1038/s41597-024-04282-0

**Published:** 2024-12-23

**Authors:** Monique Denissen, Bernhard Pöll, Kay Robbins, Scott Makeig, Florian Hutzler

**Affiliations:** 1 Paris-Lodron-University of Salzburg, Department of Psychology, Centre for Cognitive Neuroscience, Salzburg, Austria; 2 Paris-Lodron-University of Salzburg, Department of Romance Studies, Salzburg, Austria; 3 University of Texas At San Antonio, Department of Computer Science, San Antonio, USA; 4 Institute for Neural Computation, Swartz Center for Computational Neuroscience, University of California San Diego, San Diego, USA

**Keywords:** Language, Research data

## Abstract

Experimental design in language cognition research often involves presenting language material while measuring associated behavior and/or neural activity. To make the collected data easily and fully analyzable by both the original data authors and others, it is important to have detailed information about the stimulus presentation events, including the nature and properties of the presented stimuli, using a common vocabulary and syntax. We present HED LANG, a library extension of the Hierarchical Event Descriptors (HED) event annotation schema for time series behavioral and neuroimaging data. HED LANG provides an open source, standardized vocabulary for building detailed, human- and machine-actionable annotations for language cognition datasets. It builds on existing annotation systems in linguistics and is supported by a suite of HED tools for annotating, validating, searching, and characterizing HED-tagged datasets. HED LANG is specific enough to allow event data annotation at the range of levels needed to support many current research paradigms and analyses. Both HED and HED LANG are open to community input and participation, enabling them to evolve with continuing developments in language cognition research.

## Introduction

This paper introduces HED LANG^1^, a structured vocabulary that captures the characteristics of neurocognitive language experiments in sufficient detail to make them suitable for analysis by a range of analysis tools. HED LANG^1^ is an extension of the HED (Hierarchical Event Descriptor) framework^2,3^ which includes a standardized base vocabulary, detailed syntax specification, and extensive tools for annotating, summarizing, searching, and analyzing data based on HED event annotations.

### Background in language cognition research

Understanding the nature of the brain dynamics supporting language cognition has been one of the dominant areas of research in cognitive neuroscience over the past 20 years^4^. One reason language cognition has become such an active research area is that the nature of the materials used in language experiments (e.g., words, sentences) are themselves complex, as are their full descriptions. These include, for example, the number of syllables or letters in a word, its phonemic structure, how often it occurs in written language, what part of speech it represents in the given sentence, etc. Diverse cognitive processing might be engaged by each of these parameters such that multiple processing dimensions can be studied with stimuli that are easy to present and manipulate in a lab environment. In some tasks, such as the single-word lexical decision task used in early neuroimaging studies^5,6^ and still in use today^5,6^, characteristics of experimental interest are varied across conditions, while other characteristics are tightly controlled. These types of paradigms are complemented by more complex experimental designs using, for example, whole sentences and more naturalistic, ecologically valid stimuli, including full-length narratives in the form of written stories or movies^7–9^. In these studies, many relevant stimulus parameters interact naturally to produce linguistic experience, creating a challenge for data analysis^10^. Therefore, effective analysis of data recorded in psycholinguistic experiments can greatly benefit from a systematic but conveniently usable way to record these parameters to enable analysis of any type, from classical linear contrast statistics to now rapidly evolving (nonlinear) modeling methods, including multivariate pattern analysis^11^, connectivity analysis^12^, and analysis based on deep neural networks^13,14^.

Another reason why psycholinguistics is a particularly interesting domain for developing an annotation schema is that, although language is a universal human ability, the language faculty is confronted with numerous different manifestations in the form of individual languages. These convey meaning and function in various ways (e.g., using different syntactic structures, orthographic depths, and/or morphological types). The ways in which languages can vary, and how humans are able to master any particular manifestation, remains an open question in cognitive science. It has long been assumed that there are universal principles, or at least principles that are instantiated in many (if not all) of the world’s known languages. Although this notion is the basis of many theoretical frameworks in linguistics and cognitive science, it has recently been called into question because of problems with the identification of such universal principles across the diversity of existing languages^15^. However, the existence of massive linguistic variety does not necessarily preclude the existence of universal cognitive mechanism for language processing. There is evidence that some aspects of language processing may be independent of the way in which a particular function is realized. For instance, it has been shown that readers of alphabetic and logographic writing systems show similar patterns of neural activation to written stimuli^16^. To investigate this question, researchers must be able to distinguish language cognition processes that depend on a particular language manifestation from those language cognition processes that are independent of a particular language manifestation. However, much of the existing diversity of languages has not yet been adequately sampled in cognitive science^17^. In order to include a wider range of languages, data from studies of different languages need to be interoperable, they need to be able to be integrated. To do this, we need an annotation system that allows us to annotate both the widely shared as well as the highly specific features of linguistic stimuli from different languages.

### Requirements for annotation

To adequately annotate linguistic experience, an annotation system should satisfy several requirements. First, the system must contain the terminology to describe linguistic features of interest to cognitive scientists. Second, the annotations should be applicable to the type of stimulus materials they work with. Third, the system should also be open-source and community based, to ensure anyone can reproduce research using annotated datasets, and to allow the system to be updated to use the latest domain terms and concepts. Lastly, the system should be designed to allow for analysis with minimum need for transforming the annotation to enable analysis.

### Existing approaches

We first evaluated existing systems of language material description to determine whether they meet these requirements. The problem of annotation of linguistic material has been a subject of linguistics research^18^. This has led to the development of annotation approaches for systematically annotating stimulus material including the recommendations of the Expert Advisory Group on Language Engineering Standards^19^ (i.e., the EAGLES recommendations), the Universal Dependencies^20,21^ (UD), the Data Category Registry^22^ (DCR), the General Ontology for Linguistic Descriptions^23^ (GOLD), and the Ontologies of Linguistic Annotation^24^ (OLiA). Although many more systems have been proposed, we have focused on some of the most influential. Table 1 shows an overview of these approaches, when they were first released, and the information that can be annotated with each approach. Of these, Universal Dependencies and OLiA are the most recent. In particular, OLiA aims to provide a link between different annotation schemas by linking concepts via a top-level ontology.

**Table 1:** Formal annotation standards language.

Name	Year	Content
EAGLES recommendations^19^	1996	Morphosyntactic categories
Data Category Registry (DCR)^22^	2006	Morphosyntactic categories, syntax
General Ontology for Linguistic Description (GOLD)^23^	2003	General
Universal Dependencies (UD)^20,21^	2014	Part of speech, morphology, syntax
OLiA^24^	2007	General

Some systems focus on a particular subset of linguistic research. EAGLES and Universal Dependencies cover morphosyntactic categories, while GOLD also includes vocabulary for written elements of language and terminology for describing languages themselves. Universal Dependencies provides specific instructions on how annotations should be applied to language materials, specifying file formats and syntax. These specifications are important for the automatic processing of annotations, so that data with relevant properties can be found in data repositories and analyzed efficiently. Other approaches simply provide an annotation vocabulary and leave it to the user to associate the terms with the data in a meaningful way.

### Terminology coverage

The terminology used by cognitive scientists interested in language cognition overlaps significantly with terminology used in linguistics, since cognitive scientists study the cognitive implementation of the structures and patterns in language that are studied by linguists. However, the stimuli used in cognitive neuroscience experiments are not always comparable with the organic language material produced by native speakers and studied by linguists. Rather, to target or isolate specific cognitive processes^25^, stimulus material used in neuroimaging experiments is often manipulated. Over time, specific vocabulary for these manipulations has developed. A classic and still widely used example are *pseudowords*, phonotactically legal strings of letters not associated with any meaning or concept in a reader’s language (note that in cognitive science literature, pseudowords are frequently confused with *nonwords*, which are phonotactically nonconforming strings, an equally important, but different category). Such stimuli have been instrumental in understanding how, for example, the brain handles the conversion of orthography into phonology^26,27^. Although pseudowords may not be completely absent from natural language experience, when they appear they are usually judged as language errors of little conceptual interest to a linguist. Consequently, the terms “pseudoword” and “nonword” are not included in any linguistic annotation systems. Obviously, this limits the usability of these systems for cognitive science.

### Domain-specific requirements

In linguistics, the data to be analyzed are the linguistic materials themselves. By contrast, in cognitive neuroscience, the data are behavior and brain dynamics of humans interacting with linguistic material. This difference has implications for domain-specific annotation schemas. Linguistic data are usually written or spoken, often though not necessarily in the form of complete utterances or sentences. The intention to analyze and study regularities in linguistic material is reflected in how annotations are applied in specific linguistic systems. Universal Dependencies, for example, uses an adapted version of the tabular CoNLL-X format^28^ in which each row represents a word unit. The schema is designed for annotating word-in-context dependency trees and syntactic structures in sentence data. While linguistic material in the form of sentences is also used in cognitive neuroscience, language-oriented neuroimaging experiments often use single words, word lists, or language-related character strings. Therefore, it is difficult to directly use CoNLL-X to annotate stimuli used in such experiments. Instead, a suitable schema should be flexible and allow for annotation of linguistic units at any level, including the single-character, syllabic, and phonemic levels.

### Open source and community-based

The terminology used in any field is subject to change and extension. For a standardized annotation system to serve its research community it needs to be open to updates and changes from the community. Most systems developed for language annotation fulfill this requirement, as they were built using community input and are open to changes proposed from within the language research community. However, GOLD has been deprecated and is thus no longer open to input, and EAGLES recommendations, though developed over time by an expert advisory group, published its final recommendations in 1996. To ensure that any data annotations can be understood and used by anyone, standards should be formally specified and be made open source and freely available as is the case for the annotation systems in Table 1 (excepting DCR).

### Integration of metadata for analysis

The analysis of neurocognitive data is a complex, multi-step process. In the case of language cognition experiments, nearly all analysis requires detailed linguistic metadata to describe the language stimuli used in the experiments. Neuroimaging data are collected while participants perform neurocognitive tasks involving language perception (and/or production) and cognition. Typically, experiment participants are presented stimuli and asked to perform certain actions in relation to them. The neuroimaging data collected are then analyzed to assess brain dynamic correlates of participants’ experience and behavior. This requires knowing the precise nature of the events that occurred during the task experiment as well as exactly when they occurred during the data recording. For language-task experiments, not only the linguistic stimulus metadata, but also characteristics of their presentation are important – for example, whether a word was presented subliminally, whether it was an attentional target or distractor, etc. This metadata information needs to be combined to identify and assess relevant neuroimaging data time points. Other technical metadata including imaging sampling rate, etc., needs to be entered into the analysis, which must integrate all these types of metadata. Of course, this is not something that a language annotation system can do in isolation. Effective metadata integration into the larger context of the overall workflow and analysis goals requires an integrated metadata annotation system and software tool infrastructure.

### Analysis workflows and infrastructure

Most analysis tools require tool-specific metadata formats. If researchers have the metadata available in another format that is understandable to them, they can, in principle, recode the information to pass it to the analysis software in its required format. However, the open science movement advocates that data should not only be transparent to the original data authors, but thereafter to other researchers^29^. Specifically, they should be able to work with the data with little or no need for extensive research on its particular data format followed by custom reformatting to fit analysis tool expectations. This has led to a push for more standardized data and metadata organization in cognitive neuroscience and beyond, based around the FAIR principles.

### The FAIR principles

The FAIR principles were introduced as a set of guidelines for making scientific data Findable, Accessible, Interoperable and Reusable^30^. The need for machine-actionability is a keystone of the FAIR principles, requiring that data should be organized in such a way that it can be automatically identified and processed with minimal human intervention. This is particularly important given the exponential growth in the amount of data being collected and made available for reuse by the public or by accepted collaborators, and the increasing interest in processing ever larger amounts of data using artificial intelligence (AI) approaches. It would be impossible for an individual researcher to effectively search for and extract data from large data archives if this required them to first read and understand dataset format descriptions, which furthermore might often prove ambiguous or incomplete. For machines to facilitate these tasks, format standardization is essential. Some data and metadata formats (such as author names, publication dates, etc.) are routinely standardized across domains. But data are acquired in a domain-specific context, and researchers interested in reusing the data do so in a domain-specific manner. In psycholinguistics, for example, a researcher might be interested in finding data from tasks in which nouns and verbs were presented in isolation. The FAIR principles recognize the need for domain-specific data and metadata standards and vocabularies to achieve data interoperability and reusability.

In support of these principles, an infrastructure around data standards has been developed to support the automated handling of important metadata in cognitive neuroscience.

### The brain imaging data structure

The Brain Imaging Data Structure (BIDS)^31^ has formalized directory structure and file and variable naming standards for several types of neuroimaging data and associated metadata. These standards have proven to be foundational for establishing reproducible workflows for cognitive neuroscience data search and analysis, including researchers who were not involved in the original data collection – as well as by data authors or their students who may wish to further process their previously collected data. The BIDS specifications, now widely adopted, serve datatypes including fMRI, EEG, MEG, PET, and iEEG, with extensions to several other modalities now in progress. BIDS makes available shared datasets efficiently discoverable by researchers interested in a specific type of neuroimaging data. In addition, the BIDS specification provides metadata standards covering technical information about the acquired data, allowing automated extraction of essential technical parameters relevant to data analysis (for example, fMRIprep^32,33^, EEGLAB^34^, etc.).

Use of BIDS data archiving standards in many laboratories and now several public data archives increases the reusability of stored and shared data. However, as mentioned earlier, further analysis and thus full reusability of shared data requires additional information. For example, a language cognition researcher might be interested in finding datasets in which both nouns and verbs were presented in isolation during the experiment. BIDS provides a basic structure for storing event information metadata with the collected neuroimaging data. However, its formalization is limited. The BIDS recommendations specify only that event information, if present, should at minimum consist of event onset times, durations, stored in a tabular file for each data recording. Valid BIDS datasets may have no event descriptions at all. BIDS also allows inclusion of as many free descriptors for each event as a researcher wishes to add. Importantly, however, BIDS does not specify a controlled vocabulary or syntax for describing these events - any additional descriptors are optional and unstructured. Thus, formal and sufficiently detailed descriptions of the nature of the recorded events, essential for understanding and analyzing participants’ cognitive state and behavior during data collection, is outside the scope of BIDS.

### Hierarchical event descriptors

To address the need for detailed event descriptions, the Hierarchical Event Descriptors (HED) system has been developed^2,3^. The HED schema defines a basic vocabulary and syntax for describing experiment events. Terms in the HED vocabulary, (‘*HED tags’)*, can be combined into comma-separated lists (‘HED strings’) that document the nature of individual events. The HED annotation vocabulary and syntax have been formally accepted as a BIDS extension, meaning that these annotations have designated places in BIDS datasets and, when present, are validated by HED validation software as part of a BIDS validation process. HED annotations provide human-readable and machine-actionable annotations of the natures of experiment events, and as such are complementary to BIDS as they serve a need that BIDS itself does not. Terms in the standard HED vocabulary or *schema* (the HED Standard schema) cover categories broadly relevant to experiments involving human perception, action, and cognition. However, it does not include technical linguistic descriptors relevant to research in language cognition. Rather than adding all possible relevant terms in all research subfields to the HED standard schema, HED provides an extension mechanism for research communities to encapsulate annotations for domain-specific event descriptions into *HED library schemas* that can be seamlessly integrated into the Standard schema and thus the overall HED annotation system by automated HED system software tools.

Based on the requirements for annotation of language-related neurocognitive data, we have built *HED LANG*^1^, a HED library schema extension comprising a standardized terminology for annotating behavioral or neurocognitive language research experiments. Development of HED LANG^1^ has been based on existing work in linguistics which, although difficult to adopt directly, represents significant understanding of linguistic terminology and its interconnections. Additionally, we include terminology specific to language cognition research. The following Results section gives a brief overview of the terms in the LANG schema. Full details are documented in the schema itself (available online at https://github.com/hed-standard/hed-schemas/tree/main/library_schemas/lang). Next, we evaluate how well the schema succeeds in describing recent research in psycholinguistics by annotating language presentation events of experiments reported in three recent language cognition papers. Additionally we provide annotations for several fMRI language experiment datasets that are publicly available on OpenNeuro (openneuro.org).

## Results

Here, we describe the organization of the LANG schema, and illustrate its use. Specifically, we provide illustrations of its use to annotate some recently published work in the domain of language cognition. We also provide access to full HED annotations for several datasets that are publicly available on OpenNeuro. In the following section, all terms that are part of HED standard or LANG are in italics.

### LANG structure

LANG consists of over 250 tags embodying terms used to describe linguistic material. The tags are organized hierarchically, meaning that each tag is a subtype of another, more general tag. LANG tags belong to one of five categories: (1) language names (e.g., English, German, Chinese), (2) language items (e.g., morpheme, radical), (3) language item properties (e.g., grammatical categories or lexical roles), (4) language properties (e.g., orthographic depth), and (5) linguistic relations (e.g., agreement, semantic relatedness). The tags, their definitions, and their places in the HED hierarchy can be explored using the convenient online HED schema viewer (https://github.com/hed-standard/hed-schemas). Tags from the schema are combined in HED strings to describe language presentation or response events.

**Fig. 1:**
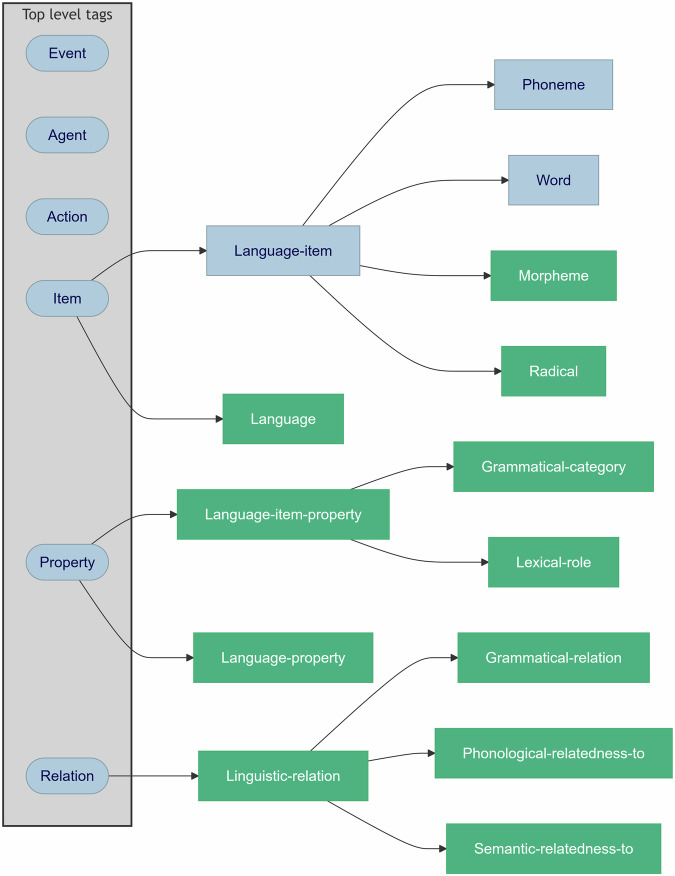
Summary overview of the embedding of a few of the LANG schema tags (green) under three top-level Standard schema tags (blue).

LANG is a ‘partnered’ HED schema, meaning it is designed to be used with the HED Standard schema vocabulary to provide a complete annotation of experiment events experienced and/or produced by an experiment participant. The overall structure of the LANG schema and its integration into the HED Standard schema are shown in Fig. 1. The Standard schema consists of six top-level tags (shown in the gray box of Fig. 1): *Event*, *Agent*, *Action*, *Item*, *Property*, and *Relation*. These top-level tags are the basis for organizing event information, covering general concepts and relations applicable across a range of domains. LANG does not add top-level tags to HED. Instead, it extends three top-level tags: *Item*, *Property*, and *Relation*. Thereby, the LANG language-related terminology is anchored in top-level Standard schema HED tag categories. This language-related terminology is represented in the green boxes in Fig. 1. The Standard schema already contains some language items, such as *Phoneme* and *Word*, which are represented by the blue boxes. Only a few LANG schema tags are shown in the figure for illustration. In total HED LANG^1^ adds over 250 language-related tags to the Standard schema.

### Example tags and usage

In this section, we give examples of tags added by LANG to the Standard schema. We explain why they are needed, and show how they can be used to describe language experiment stimuli. An important note here, is that HED is designed to describe experimental events. However, the nature of HED LANG^1^ and the field it covers means it is mostly suited to the annotation of experiment stimuli. This is also the focus of the examples we provide below. To represent a HED event these annotations would generally be accompanied by additional tags from the Standard schema, specifically *Sensory-event*, *Visual-presentation* or *Auditory-presentation*, etc.

### Language-item annotation

In the HED Standard schema, several basic, language-related tags are already defined under the tag *Language-item*, e.g., *Word*, *Phrase*, *Sentence*, and *Phoneme*. Using these tags, a presented word stimulus can be split into its relevant parts, for instance, its phonemes (phonetic elements). In linguistics this is known as parsing. It serves as a first step in annotation, defining the word as a phonetic string of elements to be described. In HED, parsing can be achieved by nested grouping of tags within a HED string using parentheses. For example, the word “reusing” can be annotated phonetically as follows (here arrayed on several lines for readability):

*(Word*, *ID/reusing*,

*(Phoneme*, *ID/r)*,

*(Phoneme*, *ID/i)*,

*(Phoneme*, *ID/j)*,

*(Phoneme*, *ID/u)*,

*(Phoneme*, *ID/z)*,

*(Phoneme*, *ID/ɪ)*,

*(Phoneme*, *ID/ŋ))*

Another area of interest in psycholinguistics is morphological processing – the perception and processing of distinct meaningful elements within some words. The technical term “morpheme”, an individually meaningful or functional unit, is not included in the Standard schema. The LANG schema adds the tag *Morpheme* as a type of *Language-item*. For example, here the word “reusing” can then be parsed into the morphemes “re”, “us[e]” and “ing”.

*(Word*, *ID/reusing*,

*(Morpheme*, *ID/re)*,

*(Morpheme*, *ID/us)*,

*(Morpheme*, *ID/ing))*

In the same vein, the LANG schema adds many other terms, such as *Radical* (a part of a Logographic character), and *Bigram*, used in some language cognition studies.

### Language-item-property annotation

Parsing presented language items is only a first step in annotating language stimulus presentation events. More important to many studies are the underlying properties of these items. For example, is the word a *Noun*, or a *Verb*? Is the *Morpheme* free or bound? The LANG library schema extension adds terms for a variety of properties that can be associated with a *Language-item*. Language item properties can be grouped in a HED string with any *Language-item*, such as *Morpheme*, or *Word*, to provide additional detail about the properties of the *Morpheme* or *Word*. Thus LANG supports parsing words into morphemes and listing properties of these morphemes that may be of use in analysis of the experiment data. In the next section we illustrate doing this for each LANG-supplied property type and show how they can be combined in a single annotation.

**Fig. 2:**
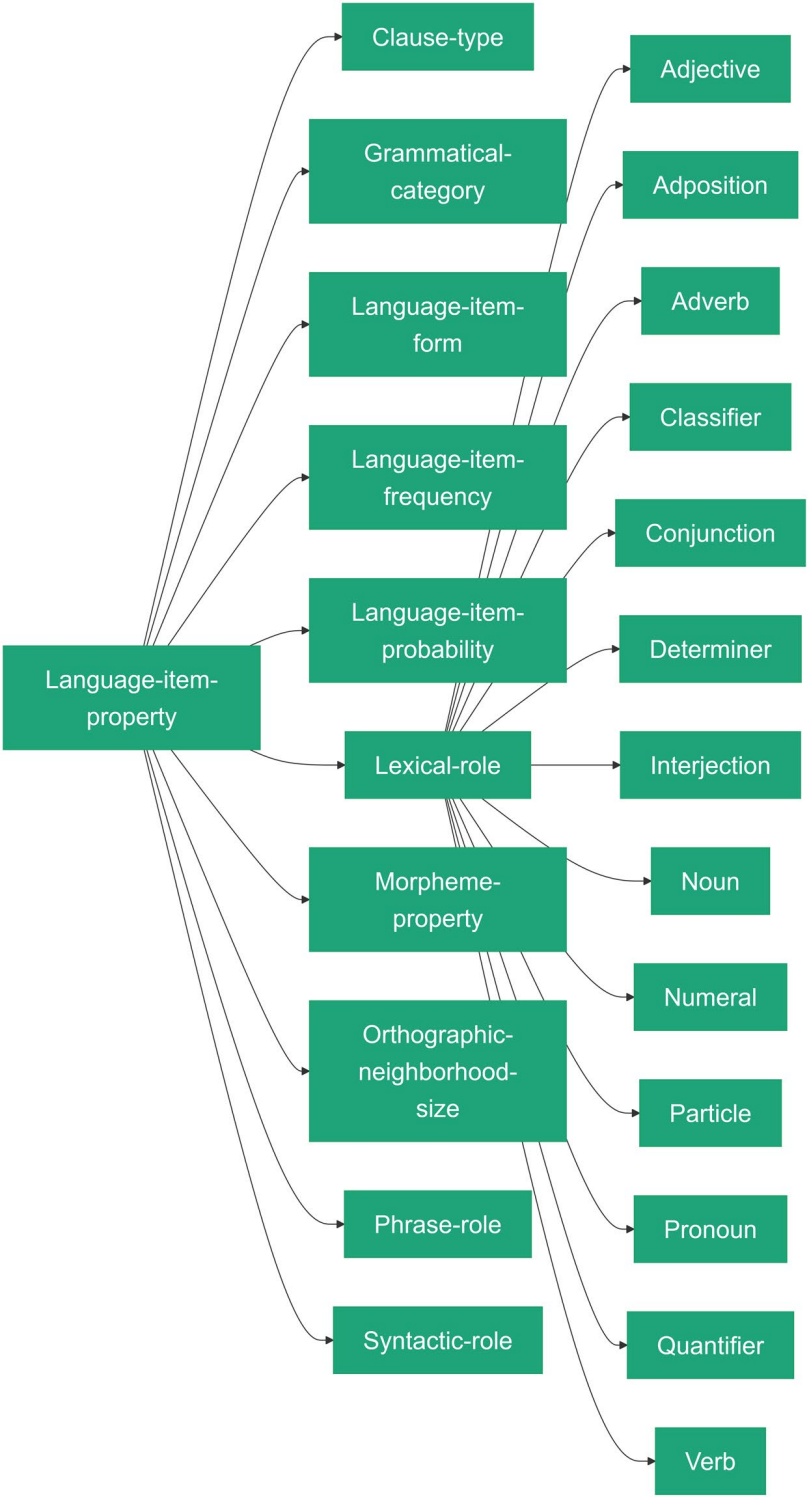
Added language item properties. Summary overview of the language item properties in HED LANG. To examplify the additional tree structure we also show the first level of categories added under Lexical-role.

The main LANG property categories are *Morpheme-property*, *Lexical-role*, *Syntactic-role*, and *Grammatical-category* (Fig. 2). Some properties are specific to one type of *Language-item*. For example, *Morpheme-property* collects tags that can be used to further describe the characteristics of a *Morpheme*. However, tags under *Lexical-role* (commonly referred to as ‘part of speech’ or ‘word class’) are in most languages a *Word* property, but in other languages may also be a property of a *Morpheme*^35^.

HED does not restrict which properties can be grouped with which items. Thus, we can add more information to the previous annotation, e.g., specifying that “reusing” has the lexical role of a verb:

*(Word*, *ID/reusing*, ***Verb***,

*(Morpheme*, *ID/re)*,

*(Morpheme*, *ID/us)*,

*(Morpheme*, *ID/ing))*

The *Grammatical-category* property contains tags about the grammatical category that a *Language-item* takes, such as *Tense*, *Countability*, and *Case*. These properties are often determined through morphology, and we recommend annotating these properties in combination with the *Morpheme-function* property. Our previous example word “reusing” is a verb with a progressive morphology through conjugation, as marked by the morpheme “ing”).

*(Word*, *ID/reusing*, *Verb*, ***Progressive***,

*(Morpheme*, *ID/re)*,

*(Morpheme*, *ID/us)*,

*(Morpheme*, *ID/ing*, *Conjugate))*

Similarly, LANG schema item *Syntactic-role* can apply to a *Word* or *Phrase* and allows the description of the syntactic role a *Language-item* takes in a phrase or sentence.

Together, these properties make it possible to provide detailed annotation of the morphosyntactic characteristics of a wide range of language items. For example, a full sentence could be annotated as follows to capture information about its grammatic structure and its morpheme composition:

*(Sentence*, *ID/He was reusing his old material*.,

*(Word*, *ID/He*, *Pronoun*, ***Subject****)*,

*(Word*, *ID/was*, *Verb)*,

*((Word*, *ID/reusing*, ***Verb***, ***Progressive***, *Syntactic-head)*,

*(****Morpheme***, *ID/re)*,

*(Morpheme*, *ID/us)*,

*(Morpheme*, *ID/ing*, *Conjugate))*

*((Word*, *ID/his*, ***Possesive-pronoun****)*,

*(Word*, *ID/old*, ***Adjective****)*,

*(Word*, *ID/material*, *Noun)*, ***Direct-object)***

This example can be extended still further, for example providing morpheme and syntax information for every word in the sentence and adding information about specific phrases or clauses within the sentence.

Another *Language-item-property* is *Language-item-form*. In neuroimaging or behavioral experiments, language can be presented as written, spoken, or signed. The LANG schema includes a property to explicitly record relations between written and spoken language items, as these, for example, have the same morphosyntactic characteristics. It also includes terms used only in cognitive science, such as the *Pseudohomophone-form*, a written form of a known word that does not follow the formal rules of an orthographic system. Pseudohomophone presentations enable language cognition researchers to distinguish processes supporting orthographic processing from processes involved in phonological and semantic processing during word recognition^36^. Pseudohomophone stimuli, are annotated as a form of word, allowing them all the characteristics associated with words (including semantic content), while marking their orthographic deviance.

Other added properties, such as *Language-item-frequency*, *Language-item-probability*, and *Orthographic-neighborhood-size*, can take on numeric values. Language item frequency has proven impact on word recognition^37^, while language item probability is often relevant in analysis of data collected in sentence processing studies^38^.

### Language and language property annotation

The LANG schema includes an extensive set of language names under the *Item* tag *Language*, organized by language family. Correspondingly, *Language-property* reflects language level properties such as *Morpheme-type*, and *Orthographic-type*.

### Linguistic-relations

The HED LANG schema^1^ contains the tag class *Linguistic-relation* comprising tags *Grammatical-relation*, *Semantic-relatedness-to*, and *Orthographic-relatedness-to*. *Grammatical-relation* currently only defines an *Agreement-to* relation, used to indicate whether two words in a sentence or phrase have grammatical agreement. In our previous example we can add a relation between the sentence subject and verb, to indicate their agreement on *Grammatical-number*:

*(Sentence*, *ID/He was reusing his old material*.,

*(Word*, *ID/He*, *Pronoun*, ***Subject****)*,

*((Word*, *ID/was*, *Verb)*, *((Agreement-with*, *Grammatical-number)*,

*((Word*, *ID/reusing*, ***Verb***, ***Progressive***, *Syntactic-head))****)***,

*(****Morpheme***, *ID/re)*,

*(Morpheme*, *ID/us)*,

*(Morpheme*, *ID/ing*, *Conjugate))*

*((Word*, *ID/his*, ***Possesive-pronoun****)*,

*(Word*, *ID/old*, *Adjective)*,

*(Word*, *ID/material*, *Noun)*, ***Direct-object****)*

Relatedness relations are important for experiments in which participants are expected to make judgements about relationships between presented words, and/or for applying Representational Similarity Analysis^39^. For each form of relatedness, field-defined measures are also available. For example, to describe degrees of orthographic relatedness LANG provides tags to record *Orthographic-Levenshtein-distance* as well as *Orthographic-Hamming-distance*.

### Usability in current research

To ensure that most or all terminology necessary for neurocognitive language research is included, we tested whether the LANG schema can support annotation of some current research in language cognition. Specifically, the LANG schema needs to support the annotation of experimental conditions. When annotations capture information that distinguishes experimental conditions, they can be used to automatize analysis pipelines. To estimate the capacity of the LANG schema in this regard, we annotated the experimental conditions reported in some recently published work. We randomly selected three papers from recently published work in three journals focused on language cognition research, specifically, studies of orthographic, morphemic, syntactical and grammatical processing, and their interactions.

### Annotating published experiments: I. Structural priming

A behavioral experiment by Van Gompel *et al*.^40^, investigated the effect of structural priming. Structural priming occurs when participants are provided with a full sentence using a specific syntax structure and are then asked to complete a partial target sentence that is designed to be completed by participants using the same syntax structure as the primed sentence^40^. The authors investigated how this priming effect is affected by repeating the sentence subject. For example, if the priming sentence contained the syntactic subject, “the farmer”, the target sentence could either repeat this subject, or else a different subject, “the seller”. Additionally, there were two priming conditions (direct, indirect) based on whether the transitive verb in the sentence is immediately followed by a direct object, as in, “The farmer gave the new potatoes …” versus an indirect object, as in, “The farmer gave the potential buyer the new potatoes …”. This resulted in two factors in the experimental design, the first was the syntactic structure of the priming sentence, which was either called a prepositional object structure, meaning the transitive verb was directly followed by the direct object, or a double object structure, meaning the transitive verb was directly followed by the indirect object. The second factor was the repetition of the noun. Table 2 shows how these conditions could be captured in HED strings.

**Table 2:** Conditions in a behavioral experiment from Van Gompel *et al*.^40^ Bolded items are discussed in the text.

Condition	HED string
***Prepositional object structure***, ***noun repeated***	*Visual-presentation*, ***Priming***, *(Sentence*, *(Phrase*, *Subject*, *(****Equal-to***, *Target)*, *(Word*, ***Transitive-verb****)*, *(Phrase*, ***Direct-syntactic-object****)))*, *(Target*, *(Phrase*, *Subject*, *(****Equal-to***, *Priming)))*
***Prepositional object structure***, ***noun not repeated***	*Visual-presentation*, *Priming*, *(Sentence*, *(Phrase*, *Subject*, *(****Not-equal-to***, *Target)*, *(Word*, ***Transitive-verb****)*, *(Phrase*, ***Direct-syntactic-object****)))*, *(Target*, *(Phrase*, *Subject*, *(****Not-equal-to***, *Priming)))*
***Double object structure***, ***noun repeated***	*Visual-presentation*, *Priming*, *(Sentence*, *(Phrase*, *Subject*, *(****Equal-to***, *Target)*, *(Word*, ***Transitive-verb****)*, *(Phrase*, ***Indirect-syntactic-object****)))*, *(Target*, *(Phrase*, *Subject*, *(****Equal-to***, *Priming)))*
***Double object structure***, ***noun not repeated***	*Visual-presentation*, ***Priming***, *(Sentence*, *(Phrase*, *Subject*, *(****Not-equal-to***, *Target)*, *(Word*, ***Transitive-verb****)*, *(Phrase*, ***Indirect-syntactic-object****)))*, *(Target*, *(Phrase*, *Subject*, *(****Not-equal-to***, *Priming)))*

HED LANG^1^ allowed encoding whether the Priming Subject was the same as the Target Subject. Note that the terms *Priming*, *Target*, and the equality relations *Equal-to* and *Not-equal-to* belong to the HED Standard schema. Combining language-specific terminology from LANG with vocabulary in the HED Standard schema provides resources researchers can use to annotate a wide range of experimental conditions.

In their analysis, Van Gompel *et.al*.^40^ assessed the proportion of responses in which the syntactic structure of the participant’s response matched that of the priming sentence, in other words, the proportion of responses in which the priming may have had an effect. They compared this proportion between conditions in which the noun in the prime sentence was either repeated in the target sentence or not. Using the HED annotation, this analysis can be efficiently reproduced.

### Annotating published experiments: II. Morphological processing

The second study we annotated, by Cayado *et al*.^41^, investigated how morpheme position affects the priming effect based on a behavioral measure, participant response time. Different theories of the development of infixation—the insertion of a morpheme within another morpheme—have led to various predictions about how infixes are processed cognitively. One model suggests that infixes may not undergo the same early, automatic processing as do prefixes and suffixes.

In Tagalog, an Austronesian language, infixes are used to indicate the perfective aspect. This feature was used by Cavado *et al*.^41^ in a priming experiment investigating whether the speed of processing of infixes differs from that of prefixes and suffixes. Participants were presented with priming stimuli consisting of a word whose morpheme either matched the morpheme of the target word or not. There were also control conditions involving semantic and orthographic priming. In all, the experiment comprised the ten conditions described and annotated in Table 3.

**Table 3:** Conditions in experiment from Cayado *et al*.^41^ Bolded items are discussed in the text.

Condition	HED string
Primed with morphological relevant prefix	Priming, Word, (Morpheme, Prefix), (**Morpheme**, (**Equal-to**, Target))
Primed with morphological irrelevant prefix	Priming, Word, (Morpheme, Prefix), (**Morpheme**, (**Not-equal-to**, Target))
Primed with morphological relevant suffix	Priming, Word, (Morpheme, Suffix), (**Morpheme**, (**Equal-to**, Target))
Primed with morphological irrelevant suffix	Priming, Word, (Morpheme, Suffix), (**Morpheme**, (**Not-equal-to**, Target))
Primed with morphological relevant infix	Priming, Word, (Morpheme, **Infix**), (**Morpheme**, (**Equal-to**, Target))
Primed with morphological irrelevant infix	Priming, Word, (Morpheme, **Infix**), (**Morpheme**, (**Not-equal-to**, Target))
Primed with semantic relevant word	Priming, Word, ((**Semantic-distance-to, Low**), Target)
Primed with semantic irrelevant word	Priming, Word, ((**Semantic-distance-to, High**), Target)
Primed with orthographic related word	Priming, Word, ((**Orthographic-distance-to, Low**), Target)
Primed with orthographic related word	Priming, Word, ((**Orthographic-distance-to, High**), Target)

Here, the dependent variable was response time to the primed conditions. Response times can be added to the event level annotation as *Agent-action* with a delayed onset from a *Sensory-event*.

*((Sensory-event*, *Visual-presentation*, *(Priming*, *Word*, *(Morpheme*, *Prefix)*, *(Morpheme*, *(Equal-to*, *Target))))*, *(Agent-action*, *Participant-response*, ***Delay/0.354****))*

Using the HED Remodeler events can be grouped by condition, facilitating scripting of statistical analyses.

### Annotating published experiments: III. Orthographic processing

We annotated a study by Fernández-López *et al*.^42^ investigating how the speed of orthographic processing is affected by rotating presented single letter stimuli. The experiment consisted of a lexical decision task in which seven-letter words were presented whose individual letters were rotated to different extents with respect to the screen. The study investigated how reaction times were affected by letter rotation and word frequency (categorized as *High* or *Lo*w). In order to demonstrate the possibilities with LANG, we added dummy data for individual word frequencies to the example annotations. While in previous examples we provided examples for each experimental condition, here we only include examples varying the rotation angle (Table 4).

**Table 4:** Conditions of experiment in Fernández-López *et al*.^42^. Bolded items are discussed in text.

Conditions	HED string
Variable angle of rotation, low word frequency	(Word, (Letter, **Rotated**, Angle/0, Item-count/7),(Word-frequency/2.58, **Low**))
Variable angle of rotation, high word frequency	(Word, (Letter, **Rotated**, Angle/45, Item-count/7), (Word-frequency/3.50, **High**))
Variable angle of rotation, pseudoword	(Pseudoword, (Letter, **Rotated**, Angle/22.5, Item-count/7))

These detailed example annotations using LANG vocabulary show that the schema can be used to annotate specific details relevant to the experimental conditions in nearly all cognitive language studies. Researchers can thus use HED annotations directly to build scripts to analyze their data. Existing schemas for language annotation lack several of the instrumental concepts required to annotate the three experiments described above. Here, we selected studies based on specific subcategories. It is important to note that studies outside of these subdomains can also be annotated using the current schema, and this increases the reusability of a dataset. For example, a study investigating metaphor processing will likely present phrases, including nouns and verbs, which may consists of individual morphemes, that can all be annotated with the current release of HED LANG^1^. However, the level of detail might not be sufficient to distinguish among specific experimental conditions.

## Discussion

Understanding the neurocognitive basis for language use and comprehension is one of the fundamental goals of cognitive neuroscience. HED LANG extends the vocabulary of the HED Standard schema based on existing linguistics data annotation systems and is tailored to the needs of language cognition researchers. The HED LANG extension will allow researchers to annotate language stimuli used in language cognition experiments in a standardized, human-readable, and machine-accessible way, ensuring that neurocognitive data based on language tasks can be readily reused for further analysis. Its structure as a HED library schema ensures detailed searchability of HED-annotated language experiment datasets. Here, we demonstrated that the HED LANG schema can support the annotation of details that distinguish experimental conditions in recently published language cognition studies. HED annotation using HED LANG^1^ enables increasing levels of automation of neuroimaging analysis to support ongoing development of language cognition research including result replication, further intensive processing, and/or extensive machine learning modeling.

In contrast to existing approaches to linguistic annotation, HED LANG^1^ meets the specific needs of cognitive scientists and neuroscientists. It not only includes basic linguistic terminology, but also terminology specific to cognitive linguistics, e.g., terms such as *Pseudohomophone-form*, *Orthographic-neighborhood*, etc. HED and HED LANG^1^ support annotation that is readily extensible to several levels of precision and granularity. HED support for nested tag groups makes it possible to annotate properties of a word, and of its constituent parts (morphemes, syllables, letters, phonemes). HED LANG^1^ is open source and, open for community input, enabling it to remain up to date with latest research domain developments. Importantly, because HED is fully integrated into existing neuroinformatics infrastructure including BIDS, cognitive researchers can use HED annotations together with existing community-based data search and processing tools. By fulfilling these requirements, HED LANG^1^ is capable of making basic contributions to ongoing progress in both behavioral and neuroscientific language cognition research.

### Current developments in language cognition research

Language cognition experiments have long been characterized by the use of tightly controlled stimuli designed to isolate the effects of specific cognitive processes on participant behavior and/or brain activity^4,43^. Although this research has led to a broad understanding of how humans process and produce language, more recently there has been increased attention to expanding the reach of cognitive language research to more ecologically valid conditions^10^, as well as across a broader sample of spoken and written languages^17,44^. These developments are important for advancing language cognition research, but they also present new challenges for data collection, annotation, and analysis. HED LANG^1^ and its associated tools, in tandem with open science practices in general, provide a solid basis for addressing these challenges.

### Adopting naturalistic paradigms

The transition to use of naturalistic stimuli in language cognition research is based on recognizing the need to understand language processing under more natural (ecologically valid) conditions^10^. However, one of the problems with analyzing data involving natural linguistic stimuli and/or responses is the presence of uncontrolled stimulus correlations^10^. Using standard statistical methods, to measure the effect of any variable of interest it is necessary to model the effects of any other present and potentially confounding variable. Information captured in HED LANG^1^ annotations can form a foundation for building such models. HED LANG^1^ terms cover language properties at a range of granularities, from individual phonemes or characters to sentences. With the HED Remodeler tool, HED annotations can provide stimulus-related information directly to analysis software such as FitLins^45^ and BIDSpm^46^, to build regressors for a general linear model, or to find and extract relevant data epochs in EEGLAB^47^. The combination of these tools can facilitate the processing of datasets involving either highly controlled or naturalistic language stimuli.

In addition, HED LANG^1^ can address the problems arising from the use of uncontrolled stimuli by further enabling the reuse of shared data. For many datasets, regressing out confounds may not be sufficient to isolate effects of interest. Potential solutions to this problem, such as providing more stimulus material^10^, are not always feasible given time and funding constraints. Taking advantage of the open science movement, stimulus set size may be increased by supplementing acquired data with data using naturalistic stimuli collected in other studies and/or laboratories. However, analysis of shared data — especially analysis across multiple datasets — presents technical interoperability challenges^48^. The HED standardized approach to annotation of experimental event and event design features is particularly important for researchers engaged in cross-study analysis. Since HED is anchored in widely accepted standards (BIDS, NWB) and has a standardized and formally specified syntax, data processing across multiple datasets can be automated effectively.

### Diversifying language cognition data

HED LANG^1^ can also help to address the problem of undersampling of many spoken and written languages. Although the goal of cognitive neuroscience is to understand the language faculty in general, the reality is that the majority of all such research is conducted on English speakers. Up to 90% of cognitive science studies are conducted by English-speaking researchers using participants speaking either English or one of a limited number of other European languages^17^. From a linguistic point of view, it is challenging to create a general annotation schema as languages differ in the ways they represent information and may have different morphological or syntactic features with no equivalent features in English or its closely related languages. Writing systems may also differ across languages, both in the visual features that distinguish symbols and in the linguistic units represented by those symbols. The way in which phonemes are represented by characters also differs among alphabetic scripts, and these differences can affect how developmental disorders manifest, even between language pairs such as German and English, that otherwise share many features^49^. Other dimensions along which writing systems can differ, include the number of elements in a script and their visual complexity^50^. To understand language as a universal human faculty, we cannot ignore these variations. Instead, they must be actively explored, requiring collection of more data from speakers of languages other than English. At the same time, we should ensure that data representing samples of under-represented languages is made more widely available in a searchable and reusable format. To facilitate this, HED LANG^1^ builds on existing work in linguistics that attempts to actively address language variations. Specifically, OLiA, which is designed to interface between different annotation schemas, some supporting specific languages^24^, and GOLD, which was an early attempt to come up with a general annotation system^23^. By building on these systems we use terminology that has been used across languages. On top of this, the structure of HED LANG^1^, with properties that are separate from the items they apply to, allows for the schema to be extended for a specific language or group of languages. For example, currently HED LANG^1^ already contains terms for *Radical*, and *Mora*, the first being an element of a logographic sign and the latter being a phonological timing unit that is relevant in some spoken languages. Additionally, we have added language-level properties to HED LANG^1^ to enable researchers to find features of interest in minimally sampled languages. In brief – HED LANG^1^ annotation can make existing as well as newly collected datasets more easily findable, searchable, and reusable.

### Current limitations

The work presented in this manuscript represents the first release of the HED LANG^1^ schema. The current version has some limitations, which we discuss here along with how they might be addressed in the future.

### Completeness

Although HED LANG^1^ adds significant vocabulary related to language cognition, it does not comprehensively cover all the topics being studied in this domain. Rather, we have focused on providing detailed coverage of these subdomains: orthographic processing, morpheme processing, syntactic processing, and grammatical processing. This selection is based in part on the coverage of existing linguistic annotation systems, as well as on the expertise of the authors and their collaborators. However, by having an orthogonal design separating linguistic units from their properties, HED LANG^1^ allows flexible extension in further domains such as phonology, semantics, and speech production. The current HED LANG^1^ is a first step towards a comprehensive system for text annotation of language experience including vocal production.

### Complexity

Another limitation of the current HED LANG^1^, and a current hurdle for data standards in general, is the effort required to properly and more completely annotate data. Although HED annotation may be easier to learn than many other annotation approaches (in particular, ontology-based annotation), it still requires some effort to learn and practice^51^. Especially because of the high number of dimensions that language stimuli can be described on. However, annotating more dimensions of stimuli and participant responses increases the likelihood of data finding further uses. Currently, doing this might require researchers to invest more effort in preparing their data for sharing. To support users, several helpful annotation tools have been developed, and the HED Working Group is committed to further tool development including AI-based annotation assistants.

One of the major reasons BIDS has been welcomed is that it is supported by a range of tools supporting data conversion (bidscoin^52^, heudiconv^53^, EEGLAB^54^) and data processing (for which the most successful example is fmriprep^32,33^). HED tools functionality extends to any HED library schema used in the annotation. HED tools already provide support for running analysis through existing BIDS apps^55^ including Fitlins^45^ and BIDSpm^46^. EEGLAB^54^ supports extraction of neuroimaging data epochs based on their contained HED tags. Current HED development focusses on extending these tools. However, we also see an opportunity to develop additional tools specific to HED LANG^1^. The type of information that is encoded in HED LANG^1^ is often derived from linguistic databases, which may use different data models to enable data retrieval. By linking existing language data models to HED LANG, annotation of language experiment data events can be further automated. This is precisely the purpose of OLiA^24^, to which many of the terms in HED LANG^1^ are already connected. Future work could focus on the automatic annotation of language stimuli described in existing databases using the power of linked data models^56^.This could enable researchers to simply linking the stimuli they used to a linguistic stimulus HED archive. These developments could minimize the level of effort required, while maximizing the satisfaction and potential career benefits of having collected and shared data.

### Future perspectives: scaling up data analysis

The development of event annotation and other metadata standards offers new opportunities for analysis of behavioral and neuroimaging data. Currently, the standard way to synthesize information across a large body of scientific reports such as, here, language cognition research studies, is through formal meta-analysis. Meta-analysis synthesizes the results of many studies to assess overlap among results so as to learn which effects reported in the literature are reproducible.

Meta-analysis begins with a literature search in which studies are selected based on strict inclusion and exclusion criteria. These often include criteria that define the types of cognitive tasks performed and the types of stimuli presented — information formalized by HED using the Standard and LANG schema. Searching the literature for such information is time-consuming. The HED LANG^1^ schema enables standardized annotation of this information, in turn enabling powerful data searches to determine which available datasets are relevant to a given research question^57^.

Once appropriate datasets have been selected, results are pooled to assess the degree of overlap among studies. It is important to note that the data types commonly recorded in cognitive neuroscience experiments, (most often fMRI and EEG or MEG), can be analyzed in different ways, oftentimes based on different assumptions and leading to different results^58^. It is therefore not always easy to determine what overlap — or lack of overlap between results means. This is especially true because researchers typically must rely on Methods descriptions, written at a higher level of abstraction, to determine whether some set of studies are comparable.

It has therefore been proposed that mega-analysis, the joint analysis of multiple data sets, may be a better way to synthesize information in a more or less diverse collection of experiment data^59^. However, few mega-analyses have been reported to date. We assume this is mainly due to complications involved in performing such an analysis. Even finding appropriate data to perform the analysis often involves harmonizing and curating the datasets so as to allow joint processing. This is cumbersome when metadata are not standardized, and is impossible when sufficient metadata are not available. Bigdely-Shamlo *et al*.^60^ have shown that HED can be used effectively to enable mega-analysis of EEG data. In future work, we hope to explore how, combined, the HED LANG schema^1^ and HED tool infrastructure can make mega-analysis more accessible.

## Methods

Development of HED LANG^1^ has been an iterative process based on existing work, author expertise, consultation with field experts, and study of current literature in language cognition. To properly design the schema, we first listed cognitive language research use cases, then focused development on meeting their requirements.

### Defining use cases

The HED LANG schema^1^ should enable researchers interested in the neural basis of language cognition to find and process data relevant to their interests. Researchers using HED-annotated language data are typically either searching for existing and relevant datasets available to them, collecting and annotating their own data to facilitate initial and/or later analyses and/or to share it with other researchers.

### Searching for relevant available data

A powerful feature of the HED taxonomy is its hierarchical structure that allows for fine-grained annotation while enabling flexible search criteria. This makes HED suitable for users interested in finding datasets based on general characteristics of language cognition experiments. General searches return experiments annotated with a fine level of detail. For example, *Letter-character* as well as *Logogram* are a type of *Character* so that any datasets presenting some type of written material can be retrieved irrespective of type of writing system that was used in the study.

The hierarchical structure of HED also plays a key role when it comes to bridging different levels of expertise. Although data is collected with a specific research question in mind, the same data, if made available to other researchers, can often serve other research interests outside the authors’ analysis goals. For example, for a researcher interested in differences between perceiving nouns and verbs, the distinction between transitive and intransitive verbs might not be relevant or known. Other verb distinctions might be critical in languages outside the knowledge and interest of the researcher. However, the hierarchical nature of HED means studies presenting such verb distinctions are still returned when searching for verbs.

As such the search use case led to two requirements for HED LANG^1^. First, the relationships between linguistic terms should be set up hierarchically and correctly. To ensure this we based the development of the schema on existing work in linguistics and consulted with field experts. More detailed notes on the link between HED LANG^1^ and existing work can be found in the Supplementary Material. Second, we ensured that terms at higher levels of the LANG tag hierarchies should be common linguistic concepts, with more specific (and domain-specific) terms under them.

### Annotation of data for analysis or sharing

Any researcher sharing data should want their data to be found and used, as this will reward their data collection, annotation, and sharing efforts with paper and data citations, and may bring new opportunities for collaboration. Researchers collecting new data, need to annotate the data to at least a level that makes their experimental conditions distinguishable if they are to use the annotations directly for analysis. For example, Shetreet *et al*.^61^ investigated the difference between ‘unaccusative’ and ‘unergative’ verbs. HED annotation of this distinction is then essential to enabling their planned analysis. The second use case thus creates an incentive for including highly specific terms that determine contrasts in neuroimaging experiments. To ensure the added terminology reaches the required level of depth, we tested whether HED LANG^1^ is able to annotate experimental designs in current research.

### Selection of current research

The field of language cognition research encompasses many subdomains. For instance, various studies focus on morpheme processing, sentence comprehension, syntactic processing, speech comprehension, speech production, semantic processing (including understanding of figurative speech) and orthographic processing. These research topics can be categorized in different ways, and often have specific research questions that require their own terminologies. To represent the depth of these terminologies, in constructing the LANG schema^1^ we have opted to focus on specific subsections of language research. For the initial release of HED LANG^1^, we focus on the following subdomains: orthographic, morphemic, syntactic and grammatical processing and their interactions.

The organization of the LANG schema^1^, its high-level concepts and design choices, based on existing related projects— ensures that the schema can be extended over time. Further domain-specific terms can be added via community input through the HED Working Group.

To ensure we adequately captured the depths of these domains, we took the following approach: From 2023 issues of three journals focused on neurolinguistic research – *Brain and Language*, *Language Cognition and Neuroscience*, and *Neurolinguistics –* we selected three (of 51) original research papers reporting research in the topic areas. We built HED annotations for stimuli presented in these experiments to verify whether HED LANG^1^ could adequately express the nature of and contrasts among the presented stimuli.

### Selection of datasets

To identify sample datasets to annotate, we searched for suitable BIDS datasets available in OpenNeuro. We looked for functional MRI data because of personal interest,—uses of HED LANG^1^ are not limited as to modality. We only included datasets involving single word presentations and having distinct paradigms to demonstrate LANG^1^ versatility .

**Table 5:** Links to annotated OpenNeuro datasets.

OpenNeuro dataset	Link (prefix: https://)
OpenNeuro ds001894^64,65^	10.18112/openneuro.ds001894.v1.4.2
Annotated^63–65^	10.60817/1w6d-6p86
OpenNeuro ds002155^69,70^	10.18112/openneuro.ds002155.v1.0.0
Annotated^69–71^	10.60817/7xmk-8247
OpenNeuro ds002382^66,67^	10.18112/openneuro.ds002382.v1.0.1
Annotated^66–68^	10.60817/60vy-2y39
OpenNeuro ds003126^72,73^	10.18112/openneuro.ds003126.v1.3.1
Annotated^72–74^	10.60817/58gs-as31
OpenNeuro ds004301^75,77^	10.18112/openneuro.ds004301.v1.0.2
Annotated^75–77^	10.60817/fsc3-d495

## Supplementary information

Influence of previous approaches

## Data Availability

HED LANG^1^ can be found under *library_schemas/lang* in the https://github.com/hed-standard/hed-schemas GitHub repository. The repository houses all HED schemas, including HED Standard. HED LANG^1^ can be found under ‘library_schemas/lang’. Each official release of HED LANG^1^ is also published to Zenodo at 10.5281/zenodo.13987483. The HED schema is available in three different formats: MediaWiki, XML, and Tabular. MediaWiki format is easy to read and write, but it does not adequately capture all schema metadata. The XML representation, which is exactly equivalent to the MediaWiki representation, is used by most HED tools for processing operations such as validation and searching. In order to create a formal ontological representation for HED that includes links to other vocabularies and complex metadata, a multi-file tabular representation of a HED schema was recently developed. This representation consists of multiple tabular files, where each describes a part of the schema. The most informative tabular file is the HED_lang_1.0.0_tag.tsv, which contains the added HED tags for HED LANG^1^. This format covers all metadata, including sources and related information such as Glottolog^62^ codes for the individual languages that are represented in HED LANG^1^. GitHub actions keep these representations in sync during the Pull Request process for update. Tools are also available to map the tabular representation into a formal ontology in OWL format (available elsewhere). As an extension of HED standard schema, the HED LANG^1^ is supported by the core HED tools and processing infrastructure. HED tags are validated within the BIDS validator, providing useful error messages for any problems in the HED annotation. HED also provides a suite of online tools (https://hedtools.org/hed/) including validation, template generation, conversion of spreadsheets to BIDS compatible files, and event remodeling operations, including HED search and factorization of event files based on HED annotations. Factorization allows the event data to be used directly with existing analysis tools such as FitLins^45^. These operations are also available from the command line interfaces of the HED Remodeler as part of the hed-python tools (https://github.com/hed-standard/hed-python) and in MATLAB (https://github.com/hed-standard/hed-matlab). The online HED annotation tool CTagger provides a user interface for HED annotations that will soon include active AI-based assistance. To further exemplify the use of HED LANG^1^ and to enhance the reusability of datasets made publicly available by other researchers, we have added HED annotations to five functional magnetic resonance imaging (fMRI) datasets that are publicly available in OpenNeuro. We selected fMRI datasets for experiments presenting isolated words. Four datasets included the identity of each presented word. For one of the datasets, we obtained this information from the data author. Using tags from HED LANG^1^ allowed us to extend the annotation beyond the information provided by the authors adding standardized, searchable HED format information about word characteristics. In order to add the annotations most datasets required an extension of the event files. To make the fully annotated data easy to use we have reshared parts of the datasets as permitted by the CC-0 license applied to OpenNeuro data. The annotated datasets are available on the Austrian NeuroCloud(https://anc.plus.ac.at), under the same license, along with appropriate attributions. The datasets used different modalities (presentation of visual and/or auditory words) and different languages. We have tried to cover some of the heterogeneity of existing languages, but are limited by the available datasets. Four of the datasets represent Indo-European languages, two of which presented words in English (ds001894^63–65^, ds002382^66–68^), one in French (ds002155^69–71^) and one in German (ds003126^72–74^). For non-Indo-European languages, the availability of sufficiently annotated datasets is even more limited. We include one dataset presenting words in Mandarin Chinese (ds004301^75–77^).Table 5 lists the datasets that were selected and processed, plus links to the original datasets and to the annotated versions. The annotated datasets were partially cloned. Specifically, we only cloned the data relevant to analysis of the functional imaging data, consisting of the functional and T1w images as well as the image metadata and minimal metadata to ensure dataset traceability and BIDS validity. Any other collected data, such as questionnaire data, additional anatomical images or diffusion weighted images can be retrieved from OpenNeuro. Event files that reported only trial onsets were reorganized so as to represent one experiment event per row. For example, one trial was split into, fixation, stimulus presentation, and subject response). For ds002382^66,67^ we updated task labels to follow the BIDS standard more closely. The provided task labels *LISTEN01* and *LISTEN02*, represented distinct runs of the same tasks. Because of the way metadata are associated with event data in BIDS formats, this representation makes it difficult to annotate the task. The task labels were updated to ‘listen’ and run labels were added accordingly. A full overview of the changes made to each dataset can be found in the updated readme of the annotated dataset. Additionally, the dataset curation was tracked using Git and full history is available as part of the dataset repositories.

## Code availability

No code was developed in this work.
