# Supplementary material for: HED LANG – A Hierarchical Event Descriptors library extension for annotation of language cognition experiments
Source: Sci Data. 2024 Dec 23;11:1428. doi: 10.1038/s41597-024-04282-0 (PMC11666789; doi:10.1038/s41597-024-04282-0)
Supplement: Supplementary file 1 — Influence of previous approaches [file 41597_2024_4282_MOESM1_ESM.pdf]

Supplementary Material

Influence of previous approaches

We based HED LANG on existing systemic approaches to linguistic annotation, mainly OLiA[1], Universal Dependencies[2,3] and GOLD[4].

OLiA

OLiA is a collection of ontologies and annotation models, linked through a top-level ontology. In the following comparison, we consider only the OLiA reference model, which was based in part on GOLD and the EAGLES recommendations.

As in OLiA, in HED LANG language units are separate from their properties (using the distinction between *Language-item* and *Language-item-property)*. In OLiA, however, only some language items are represented and they are not part of a single category. For example, in OLiA "Morpheme" is a type of "Morphological category" *,* while *Phrase, Sentence, Clause,* and *Fragment* are "Constituents". Because of the larger difference in structure between the existing HED terms we opted not to follow this organization. Additionally, "Morpheme" has subcategories "Affix", "Infix", and "Suffix". HED LANG contains the same concepts but as types of *Morpheme-position* property. This deviation was chosen to better maintain the orthogonal design of the HED taxonomy, since there are other bases to categorize morphemes, such as whether they are bound or free.

The *Lexical-role* in HED LANG is comparable to OLiA's "Morphosyntactic category". We opted for the LANG term *Lexical-role,* as GOLD uses "Morphosyntactic property" to group concepts like "Case" and "Countability". LANG terms under *Grammatical-category* are represented as features in OLiA that are top level categories. Because of the inconsistency in what defines a morphosyntactic property across prominent existing schemas like OLiA and GOLD, we have stepped away from the term and used *Lexical-role* for part of speech or word class and *Grammatical-category* for tense, gender, mood, etc. The content of the categories overlaps with what is reflected in OLiA, although not the same level of detail is covered.

OLiA contains a top-level "Syntactic role" which represents the same concepts as *Syntactic-role* in HED LANG, including terms for *Indirect-syntactic-object* and *Direct-syntactic-object*. These categories are widespread in the known languages of the world and are used in experiments[5]. Additionally, *Secondary-predicate* has been used in studying the basis of grammatical processing[6].

Note here that descriptive categories in the field of syntax are often closely linked to a specific theoretical approach. Specifically, some of the terms that were added under *Syntactic-role*, e.g., *Adjunct*, come from the theory of Generative Grammar. However, the wide influence of this theory means that most of these terms are generally well understood. As such their relation to theory does not restrict the general descriptive validity of the added terminology.

GOLD

The *Language-item* in HED is comparable to "Linguistic Unit" in GOLD. These are further subdivided based on whether they are "perceptual units", "grammatical units" or "syntactic units" such that *Word* is a type of "Syntactic Unit" labeled "Syntactic Word". In HED LANG we did not add this

additional classification, since we see limited benefit for search and the addition would make the schema more difficult to use for researchers without a linguistic background.

GOLD contains a type of object “Linguistic expression”, which can be spoken, written, or signed. It is meant to refer to a specific instantiation of a “Language Unit”. In HED LANG, whether a language unit is spoken, written, or signed, is reflected as a property of a *Language-item* to better accommodate the needs of the cognitive scientist. From the cognitive perspective the common properties of language items independent of expression are highly relevant to language processing.

As is the case in OLiA, “Affix” is a type of “Morpheme” in GOLD. But in contrast to OLiA, “Reduplication” is a type of “Bound morpheme”. In HED LANG both are position properties of the morpheme. In this way we can support annotation of both fusional and agglutinating languages.

GOLD has a class for “Language Varieties”, but these provide labels to classify languages based only on their status and use (e.g., whether a language is considered extinct). Although this is relevant for language research, in cognitive science we deal almost exclusively with languages currently in use, and it is more relevant to find datasets using related languages, which is why we opted to categorize by family.

The GOLD “Morphosyntactic property” corresponds to *Grammatical-category* in HED LANG. Because of the inconsistency with OLiA, we relabeled this tag. Additionally, in GOLD, there is a “Morphosemantic property” that represents those grammatical categories not affected by syntax. Again, we have opted to not follow this categorization because of limited benefit and how it would complicate the schema.

Universal Dependencies

The Universal Dependencies (UD) can be used to annotate sentence structure and certain word properties. Words can be annotated with part of speech tags as well as additional “features”. There are numerous language specific ‘treebanks’, which contain language specific tags and features, but here we focus on the universally defined terminology.

Annotation with Universal Dependencies starts with tokenization, or parsing of text into words, this is similar to what can be done using nested groups in a HED string, but because of the additional language items that are represented in HED LANG, additional levels of granularity can be annotated with HED LANG.

The universal part-of-speech tags are divided into “open class” words, “closed class” words, and “other”. We represent these concepts under *Lexical-role*. Because of the separation between open class and closed class words in UD, an auxiliary verb is not a type of verb in UD, and verb refers exclusively to content verbs. We opted to follow the organization of GOLD and OLiA instead.

UD universal features cover lexical and grammatical properties of words. They are subdivided into lexical features and inflectional features. However, it is important to note that this organization is not a strict hierarchy of terminology, these are approximate categories. The lexical features are properties such as “reflexive” and “pronoun type”. Following OLiA and GOLD, we have represented these as subclasses of specific *Lexical-role* tags, for instance, *Reflexive-pronoun* and *Possessive-*

pronoun. Part of the inflectional features are represented as *Grammatical-category* in HED LANG. This is more in line with GOLD.

Glottolog

Glottolog is a catalogue of languages, language families and dialects.[7] We included languages by language families based on Glottolog.

References

1. Chiarcos, C. & Sukhareva, M. OLiA – Ontologies of Linguistic Annotation. *Semantic Web* **6**, 379–386 (2015).

2. Nivre, J. *et al.* Universal Dependencies v1: A multilingual treebank collection. in *Proceedings of the Tenth International Conference on Language Resources and Evaluation (LREC'16)* (eds. Calzolari, N. et al.) 1659–1666 (European Language Resources Association (ELRA), Portorož, Slovenia, 2016).

3. Nivre, J. *et al.* Universal Dependencies v2: An Evergrowing Multilingual Treebank Collection. Preprint at https://doi.org/10.48550/arXiv.2004.10643 (2020).

4. Farrar, S. & Langendoen, T. A linguistic ontology for the semantic web. *GLOT Int.* **7**, 97–10 (2003).

5. van Gompel, R. P. G., Wakeford, L. J. & Kantola, L. No looking back: the effects of visual cues on the lexical boost in structural priming. *Lang. Cogn. Neurosci.* **38**, 1–10 (2023).

6. Durand-López, E. M. Working memory and L2 gender agreement processing in structural distance conditions. *J. Cult. Cogn. Sci.* (2024) doi:10.1007/s41809-024-00143-9.

7. Hammarström, H., Forkel, R., Haspelmath, M. & Bank, S. Glottolog 5.0. https://doi.org/10.5281/zenodo.10804357 (2024).
